# Computer-Aided Image Analysis and Fractal Synthesis in the Quantitative Evaluation of Tumor Aggressiveness in Prostate Carcinomas

**DOI:** 10.3389/fonc.2016.00110

**Published:** 2016-05-09

**Authors:** Przemyslaw Waliszewski

**Affiliations:** ^1^Department of Surgery, Urological Unit, Hufeland Clinics GmbH, Bad Langensalza, Germany; ^2^The Będlewo Institute for Complexity Research, Poznań, Poland

**Keywords:** fractals, complexity, grading, tumor aggressiveness, prostate, cancer

## Abstract

The subjective evaluation of tumor aggressiveness is a cornerstone of the contemporary tumor pathology. A large intra- and interobserver variability is a known limiting factor of this approach. This fundamental weakness influences the statistical deterministic models of progression risk assessment. It is unlikely that the recent modification of tumor grading according to Gleason criteria for prostate carcinoma will cause a qualitative change and improve significantly the accuracy. The Gleason system does not allow the identification of low aggressive carcinomas by some precise criteria. The ontological dichotomy implies the application of an objective, quantitative approach for the evaluation of tumor aggressiveness as an alternative. That novel approach must be developed and validated in a manner that is independent of the results of any subjective evaluation. For example, computer-aided image analysis can provide information about geometry of the spatial distribution of cancer cell nuclei. A series of the interrelated complexity measures characterizes unequivocally the complex tumor images. Using those measures, carcinomas can be classified into the classes of equivalence and compared with each other. Furthermore, those measures define the quantitative criteria for the identification of low- and high-aggressive prostate carcinomas, the information that the subjective approach is not able to provide. The co-application of those complexity measures in cluster analysis leads to the conclusion that either the subjective or objective classification of tumor aggressiveness for prostate carcinomas should comprise maximal three grades (or classes). Finally, this set of the global fractal dimensions enables a look into dynamics of the underlying cellular system of interacting cells and the reconstruction of the temporal-spatial attractor based on the Taken’s embedding theorem. Both computer-aided image analysis and the subsequent fractal synthesis could be performed effectively using the standardized software implemented on the world internet platform. This platform should help to verify the quantitative criteria for the identification of indolent prostate cancers or highly aggressive cancers as well as to test the improved statistical models for progression risk assessment within a single prospective study.

Tumor aggressiveness can be defined as a potential of cancer cells for proliferation and self-organization into structures of the higher order, such as gland-like structures as well as a local infiltration and metastasis formation. Although this parameter is defined by the evaluation of the static tumor architecture, it reveals some dynamic context spanning both the spatial and the temporal dimensions of tumor growth (1). Therefore, tumor aggressiveness plays a role in the statistical models of progression risk assessment. Accuracy of those models is about 70%. Despite the coapplication of different parameters characterizing tumor growth, tumor progression cannot be predicted by those deterministic models without uncertainty.

In the case of prostate carcinomas, pathologists developed, so far, about 40 grading systems scoring tumor aggressiveness (2). All of them are subjective, and, therefore, have one fundamental weakness, that is, a large inter- and intraobserver variability. For the Gleason score system, the variability of 40–80% and the kappa coefficient for interobserver agreement 0.15–0.7 were reported (3–5). Although the recent modification of the Gleason system, which was done the third time in the last 15 years, seems to simplify grading by combining the well-known Gleason grades into the five prognostic groups, it does not eliminate the subjective nature of the image evaluation. That novel subjective approach will most likely be burdened with both a similar variability and inaccuracy for the progression risk assessment (6). Indeed, prostate carcinomas can be classified with the highest possible accuracy into the classes of equivalence using the objective values of the global fractal dimensions [see Table 2 in Ref. (7)]. However, results of the subjective evaluation of the same prostate carcinomas do not match the results of the objective approach so perfectly [see Table 1 in Ref. (7)].

While definitions of the Gleason grades are clear and concise, the exact subjective matching by eye and mind is very difficult even for the experienced pathologist. Usually, it is easier to ascribe a score if prostate carcinoma has a homogeneous architecture with regular gland-like structures or cell infiltrates. A discrepancy between pathologists concerns mostly the borderline cases, such as those described subjectively in the previous grading system by the Gleason score 3 + 4 or 4 + 3, 4 + 5 or 5 + 4, the complex score 3 + 4 (+5), etc. Although prostate carcinomas graded so far with the score 4 + 5 and 5 + 4 were combined in 2015 into the common prognostic group, carcinomas with the Gleason score 3 + 4 and 4 + 3 are still classified in the two different prognostic groups (6). Besides an obvious problem with the above-mentioned intra- and interobserver variability, the subjective evaluation of aggressiveness in those cases is not able to provide us with some precise criteria for the identification of patients who do not need any treatment and can be monitored within a strategy of the active surveillance. In consequence, one may expect that a large number of patients will continue to undergo aggressive treatment without even knowing if they really need it to. Indeed, there are many histological details, such as density of cellular infiltration or a number, size, and geometry of pseudoglands, that cannot be evaluated by human mind and eyes quantitatively with sufficient precision. Since pathologists are not able to grade unequivocally those borderline cases, a combination of the known Gleason grades into the five prognostic groups is a fair proposal (6). Results of the cluster analysis with the coapplication of seven complexity measures characterizing the spatial distribution of cancer cell nuclei in 208 prostate carcinomas suggest that this number should be reduced even further to just three grades corresponding to low, intermediate, and high aggressive prostate carcinomas (7).

Computer-aided image analysis enables the objective, quantitative evaluation of tumor aggressiveness using the digitalized H&E images exclusively. No additional immunohistochemical staining or molecular assay is necessary. Certainly, there are a number of ways to describe tumor architecture, such as distance analysis, texture analysis, or morphometric analysis [reviewed in Ref. (8)]. Since June 10, 1854, the most important day in the history of mathematics, when Bernhard Riemann gave a habilitation lecture at the Georg-August University of Göttingen, Germany, it has been known that geometry of time-space is much more than just the static arena for physical events that occur in it (9–11). According to Riemann, dynamics of the underlying phenomena, such as cellular interactions, influences the geometry of time-space in which those cells interact and vice versa. By studying geometry, one can get information about the underlying dynamics. In the case of the interacting cellular systems, both dynamics and geometry are of fractal nature and are coupled to each other (1). Hence, fractal synthesis that is based on the principles of fractal geometry, the circular fractal model of adenocarcinoma, and a number of complexity measures characterizes well the geometry of the spatial distribution of cancer cell nuclei in tumor images. A complex image can be described by a single number, such as the value of the global capacity fractal dimension *D*_0_ (8) or by a series of the interrelated numbers, such as the global and local fractal dimensions, entropy, and lacunarity (7, 8, 12, 13). In addition, the coapplication of local fractal maps, that is, algorithms calculating the value of the local fractal dimension for each pixel in tumor image helps to distinguish between the borderline cases causing, so far, difficulties during the subjective evaluation, such as Gleason 3 + 4 or 4 + 3.

Most important, those complexity measures define some quantitative criteria that indicate unequivocally, truly low-aggressive carcinomas or high-aggressive ones (7, 12). Those criteria could not be defined using any subjective approach. Furthermore, the quantitative restratification of carcinomas according to the values of the complexity measures into the classes of equivalence is crucial for the systematic search of some morphometric features of cancer cells that may enable the evaluation of tumor aggressiveness in biopsy specimen, not just in surgical specimen. It is possible that the re-analysis of the old data banks re-stratified into the classes of equivalence according to the *D*_0_-cut off values will identify some additional geometrical parameters characterizing cancer cells that were rejected owing to the misleading subjective classification according to the Gleason criteria. Finally, the fractal dimensions enable the reconstruction of the temporal–spatial attractor of the underlying dynamic process, based on the Takens’ embedding theorem. In this way, dynamics of tumor progression in each individual case could be predicted perhaps better than by the current deterministic models.

There is a natural tendency in research to relate the quantitative approach to the known subjective grading system, e.g., by a statistical comparison of patient survival stratified according to the objective vs. subjective parameters using the Cox regression model or by a comparison of the accuracy of case classification. It should be realized, however, that the subjective and objective parameters belong to two different categories. Therefore, such comparison would be a logical fallacy. In other words, results of the subjective evaluation cannot be used as the absolute frame of reference to validate the novel quantitative approach (7, 8, 12).

Clinical studies show that the correlation between gene expression profiling or proteomic tests and stratification of carcinomas according to the subjective Gleason score is much lower than 1.0 (14). It is also likely that the novel quantitative stratification of prostate carcinomas into the classes of equivalence defined according to the values of the global capacity fractal dimension *D*_0_ or information fractal dimension *D*_1_ will not correlate well with expression of biomarkers of epithelial differentiation. Indeed, science of complexity indicates that emerging phenomena occurring at the macroscale, such as the evolution of tumor architecture from the gland-like structures, representing the higher order of self-organization and the lower complexity into cellular infiltrates, representing the lower order and the higher complexity, can be independent of the molecular events at the microscale. Second, the results of computer simulations indicate that the shift from one form of the spatial organization to the other one may be determined just by a single mutation of a critical gene, such as the ϵ-cadherin gene (12).

Since the values of complexity measures may vary depending on tumor area, pathologist should measure complexity of the spatial distribution of cancer cell nuclei in different regions and calculate the mean and median values, SDs, minimum, maximum, etc. The report with the common statistics can be easily generated. In addition, each case can be shown on the 2D- or 3D-scatter plots with the subordination to the appropriate class of equivalence called complexity class. Each prostate carcinoma can be localized unequivocally in the space of variables and compared with the other cases in the World DataBank with the known course of the disease (see Figure 1). Those multidimensional sets of parameters could be accumulated by the self-organized maps, a kind of self-educating neural network and presented graphically in a convenient manner (13).

**Figure 1:**
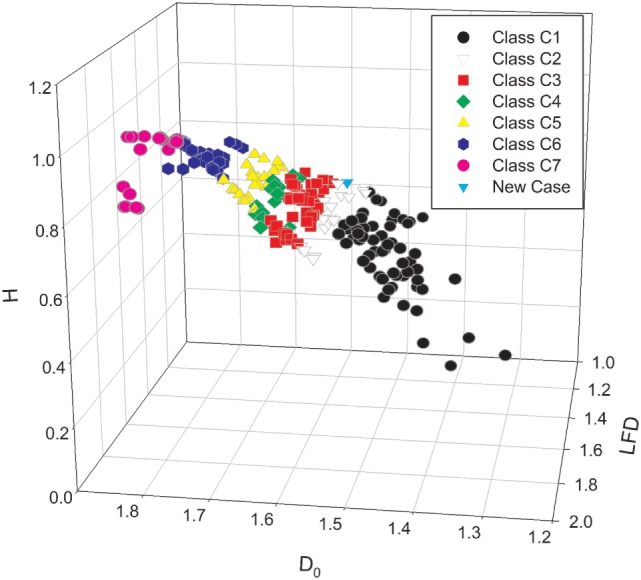
**The three-dimensional scatter plot presents the stratification of the set of prostate carcinomas with the known clinical course of disease into the seven classes of equivalence (7)**. The classes of equivalence were defined by the mean values of the global capacity fractal dimension *D*_0_. Each prostate carcinoma was characterized by a triplet of the mean values of the following complexity measures: the global capacity fractal dimension *D*_0_, the local fractal dimension LFD, and entropy H. The class C1 (black circles), C4 (green diamonds), and C7 (red circles) represents exclusive patterns with the Gleason score 3 + 3, 4 + 4, and 5 + 5, respectively. The white triangles down denote the carcinomas of the class C2. The red squares stand for the cases of the class C3. Those two classes are composed mostly of the carcinomas with the Gleason score 3 + 4 and 4 + 3, respectively. In addition, those classes contain some carcinomas classified primarily as Gleason 3 + 3 or Gleason 4 + 4 and restratified to those classes on the basis of the values of the global capacity fractal dimension *D*_0_. The yellow triangles up stand for the complexity class C5, and the black pentagons denote the complexity class C6_._ The class C5 contains mostly carcinomas with the score 4 + 5, and the class C6 has carcinomas with the score 5 + 4. The spatial distribution of cancer cell nuclei in a single new case of prostate carcinoma was characterized numerically by the same computer algorithms. The case is represented on the scatter plot as a blue triangle. Since prostate carcinomas are known to be heterogeneous, computer-aided image analysis must comprise many different regions of interest. Results of statistical analysis can comprise values, such as mean, median, minimum, maximum, and SDs. Both the mean and the maximal values of the *D*_0_ determine the class of equivalence for the given carcinoma. A difference between the integer Euclidean dimension 2.000 and the maximal value of the LFD determines the risk of metastasis formation in a reverse proportional manner.

One must emphasize here a role of the Internet that offers a technical possibility for the unified evaluation of tumor aggressiveness by different clinics in the world. Since different software may generate different values of the complexity measures, it is important to define conditions for digitalization of histological images, such as light intensity, microscope aperture, magnification, resolution, and format. Computer software on the platform would be standardized. All, what is necessary to establish such an Internet platform for the planet, is a kind of the international agreement on the software to be used, on the parameters to be measured, on a financial support to set up the platform and to run this large prospective study. This could be achieved at the level of the world urological associations, such as the EAU, AUA, or SIU, in a cooperation with the appropriate world organizations of pathologists. From the technical viewpoint, there is no problem to set up such a system. The question remains if pathologists accept computer algorithms as important supportive tools that will change both the style of their responsible work and accuracy of results. This kind of a planetary prospective project is a challenge for medical community. But, there is no other way for humanity to develop as to move frontiers continually by accepting the challenges.

## Take Home Message

Owing to some ontological and logical limitations, a comparison of results of the quantitative evaluation of tumor aggressiveness with results of any subjective evaluation is impossible. The quantitative approach based on some holistic parameters, such as the global fractal dimensions is one of the possibilities. This approach may provide more information on dynamics of tumor growth. Tools for both computer-aided image analysis and fractal synthesis implemented on a unified world Internet platform might facilitate the quantitative evaluation of tumor aggressiveness, and a verification of the quantitative criteria for the identification of indolent prostate cancer within a single prospective study.

## Author Contributions

PW: all ideas, construction of the prostate data bank, digitalization of images, statistical analyses, and writing of the manuscript.

## Conflict of Interest Statement

The author declares that the research was conducted in the absence of any commercial or financial relationships that could be construed as a potential conflict of interest.

## References

[1] Waliszewski P. A principle of fractal-stochastic dualism and Gompertzian dynamics of growth and self-organization. Biosystems (2005) 82(1):61–73. 10.1016/j.biosystems.2005.05.011 16024163

[2] Humphrey PA Grading of prostatic carcinoma. Prostate Pathology. Chicago: ASCP Press (2003). p. 338–74.

[3] Netto GJ Eisenberger M Epstein JI TAX 3501 Trial Investigators. Interobserver variability in histologic evaluation of radical prostatectomy between central and local pathologists: findings of TAX 3501 multinational clinical trial. Urology (2011) 77(5):1155–60. 10.1016/j.urology.2010.08.031 21146858 PMC3449146

[4] Egevad L Ahmad AS Algaba F Berney DM Boccon-Gibod L Compérat E Standardization of Gleason grading among 337 European pathologists. Histopathology (2013) 62(2):247–56. 10.1111/his.12008 23240715

[5] Berney DM Algaba F Camparo P Compérat E Griffiths D Kristiansen G The reasons behind variation in Gleason grading of prostatic biopsies: areas of agreement and misconception among 266 European pathologists. Histopathology (2014) 64(3):405–11. 10.1111/his.12284 24102975

[6] Epstein JI Zelefsky MJ Sjoberg DD Nelson JB Egevad L Magi-Galluzzi C A contemporary prostate cancer grading system: a validated alternative to the Gleason score. Eur Urol (2015) 69(3):428–35. 10.1016/j.eururo.2015.06.046 26166626 PMC5002992

[7] Waliszewski P The quantitative criteria based on the fractal dimensions, entropy and lacunarity for the spatial distribution of cancer cell nuclei enable identification of low or high aggressive prostate carcinomas. Front Physiol (2016) 7:34 10.3389/fphys.2016.00034 26903883 PMC4749702

[8] Waliszewski P Wagenlehner F Gattenloehner S Weidner W. On the relationship between tumor structure and complexity of the spatial distribution of cancer cell nuclei: a fractal geometrical model of prostate carcinoma. Prostate (2015) 75(4):399–414. 10.1002/pros.22926 25545623

[9] Weber HM Dedekind R Bernhard Riemann’s Gesammelte Mathematische Werke und Wissenschaftlicher Nachlass. Cambridge: Cambridge University Press (2013).

[10] do Carmo MP Differential Geometry of Curves and Surfaces. London: Prentice Hall (1976).

[11] do Carmo MP Riemannian Geometry. Boston: Birkhäuser (1992).

[12] Tanase M Waliszewski P. On complexity and homogeneity measures in predicting biological aggressiveness of prostate cancer; implication of the cellular automata model of tumor growth. J Surg Oncol (2015) 112(8):791–801. 10.1002/jso.24069 26462459

[13] Waliszewski P Dominik A Wagenlehner F Gattenlöhner S Weidner W Complexity measures and classifiers in objective prostate cancer grading. Poster Nr 741, 29th EAU Congress. Stockholm (2014).

[14] Reichard CA Stephenson AJ Klein EA. Applying precision medicine to the active surveillance of prostate cancer. Cancer (2015) 121(19):3403–11. 10.1002/cncr.29496 26149066 PMC4758404

